# Effects of Dietary Histidine on Growth Performance, Serum Amino Acids, and Intestinal Morphology and Microbiota Communities in Low Protein Diet-Fed Piglets

**DOI:** 10.1155/2020/1240152

**Published:** 2020-11-28

**Authors:** Meng Kang, Jie Yin, Jie Ma, Xin Wu, Ke Huang, Tiejun Li, Long Ouyang

**Affiliations:** ^1^College of Animal Science and Technology, Hunan Agricultural University, Changsha, China; ^2^Key Laboratory of Agro-ecological Processes in Subtropical Region, Institute of Subtropical Agriculture, Chinese Academy of Sciences; Scientific Observing and Experimental Station of Animal Nutrition and Feed Science in South-Central, Ministry of Agriculture; Hunan Provincial Engineering Research Center for Healthy Livestock and Poultry Production, Hunan, China; ^3^Hunan Provincial Veterinary Drug and Feed Supervision Institute, Changsha, China

## Abstract

Previous study showed that low protein diet-fed pigs are characterized by lower histidine concentration in the serum and muscle, suggesting that histidine may involve in protein-restricted response. Thus, the current study mainly investigated the effects of dietary histidine on growth performance, blood biochemical parameters and amino acids, intestinal morphology, and microbiota communities in low protein diet-challenged-piglets. The results showed that protein restriction inhibited growth performance, blood biochemical parameters and amino acids, and gut microbiota but had little effect on intestinal morphology. Dietary supplementation with histidine markedly enhanced serum histidine level and restored tryptophan concentration in low protein diet-fed piglets, while growth performance and intestinal morphology were not markedly altered in histidine-treated piglets. In addition, histidine exposure failed to affect bacterial diversity (observed species, Shannon, Simpson, Chao1, ACE, and phylogenetic diversity), but histidine-treated piglets exhibited higher abundances of *Butyrivibrio* and *Bacteroides* compared with the control and protein-restricted piglets. In conclusion, dietary histidine in low protein diet enhanced histidine concentration and affected gut microbiota (*Butyrivibrio* and *Bacteroides*) but failed to improve growth performance and intestinal morphology.

## 1. Introduction

Low protein diet has been widely used in pig industry to save protein sources and alleviate the nitrogen excretion [1]. Covering amino acid requirements maintains the growth performance in proper low protein diet-fed pigs [2]. For example, we found that reducing 3% dietary crude protein and balancing lysine, methionine, threonine, and tryptophan fails to markedly affect feed intake and weight gain from weaning piglets to finishing pigs, while 6% protein limitation causes a significant reduction in growth performance [3]. Also, low protein diet has been reported to enhance meat quality of growing and finishing pigs by targeting lipid metabolism, fiber characteristics, and amino acid metabolism in the muscle tissue [4]. Meanwhile, protein restriction in other animal models also shows many merits, such as improving healthy lifespan and reducing tumor incidence [5, 6]. The potential mechanism may be associated with amino acid metabolic programming, while amino acid metabolic response and the requirement in protein-restricted models have not been fully study.

Our previous study revealed a marked change in amino acid metabolism in low protein diet-fed pigs, especially for reduction in histidine and branched-chain amino acids [3]. Zhang et al. reported that dietary supplementation with branched-chain amino acids increases growth performance and muscle mass in low protein diet-challenged piglets [7]. However, little study has focused on the role of histidine in protein-restricted piglets. In addition, histidine deficiency in diet shows a negative relationship with lactational performance in dairy cows [8]. Furthermore, dietary 0.18% histidine improves muscle and blood 1,1-diphenyl-2-picrylhydrazyl radical scavenging capacity in turkey [9]. Thus, this study mainly investigated the effect of histidine on low protein diet-fed piglets with focuses on growth performance, serum amino acid profiles, and gut microbiota communities.

## 2. Materials and Methods

### 2.1. Animals and Groups

24 piglets (Landrace×large white, gibbed male, and 7.678 ± 0.17 kg body weight) were randomly divided into 3 groups (*n* = 8): a control group (17% CP), a low protein group (14% CP), and a low protein group supplemented with 0.1% histidine to keep the same concentration compared with the control diet. Data from the control and low protein groups shared with our previous study [10]. Animals were housed individually and given fresh feed 3 times per day for 35 days. At the end of each period, all piglets were killed for sample collection. Experimental protocol of piglet study was approved by the Animal Care and Use Committee of Institute of Subtropical Agriculture, Chinese Academy of Sciences.

### 2.2. Growth Performance

Feed intake and body weight from each animal were recorded. The liver, kidney, and spleen indexes were also calculated according to the ratio to the body weight of piglet.

### 2.3. Blood Biochemical Parameters

Blood samples were harvested from anterior vena cava and centrifugated (3000 rpm, 10 min, and 4°C) for serum collection. Biochemical kits for total protein (TP), albumin (ALB), alanine aminotransferase (ALT), aspartate aminotransferase (AST), alkaline phosphatase (ALP), urea nitrogen (BUN), creatinine (CREA), glucose (Glu), C-reactive protein (CERL3), bile acid (BILT3), and immunoglobulin G (IGG) were purchased from Roche (Shanghai, China) and tested by the Cobas c-311 coulter chemistry analyzer [3].

### 2.4. Blood Sample Preparation and Amino Acid Determination

18 amino acids in the serum were determined via a High-speed Amino Acid Analyzer L-8900 (Japan).

### 2.5. Intestinal Histological Examination

Middle sections of the jejunum and ileum from all animals were fixed with 4% paraformaldehyde-PBS and then dehydrated and embedded in paraffin blocks for H&E dyeing. Villus length, crypt depth, and villus surface area were measured.

### 2.6. Gut Microbiota Sequencing

Total genome DNA from distal intestinal digesta samples was extracted for amplification using specific primer with the barcode (16S V3+4). Sequencing libraries were generated and analyzed according to our previous study [11]. Subsequent analysis of alpha diversity (Observed-species, Chao1, Shannon, and Simpson) was performed basing on this output normalized data.

### 2.7. Statistical Analysis

Statistical analysis of all data was performed using the one-way analysis of variance (ANOVA) to test homogeneity of variances via Levene's test and followed with Duncan's test (IBM SPSS 23 software). Data are expressed as the mean. Values in the same row with different superscripts are significant (*P* < 0.05).

## 3. Results

### 3.1. Effect of Dietary Histidine on Growth Performance in Low Protein Diet-Fed Piglets

Final body weight, average daily weight gain, and average daily feed intake in each week during the experiment were analyzed, and the results showed that reducing dietary CP markedly reduced final body weight and average daily weight gain (*P* < 0.05), while dietary supplementation with histidine failed to improve body weight gain (*P* > 0.05). Feed intake was not altered at weeks 1-3, but low protein diet reduced feed intake from week 4 to week 7 (*P* < 0.05). Interestingly, dietary histidine markedly improved average feed intake at week 6 (*P* < 0.05) but had no effect in other weeks (Table 1).

### 3.2. Effect of Dietary Histidine on Relative Organ Weight in Low Protein Diet-Fed Piglets

The relative weights of the liver, kidney, and spleen were calculated, and the results showed that the liver relative weight was markedly increased and the kidney relative weight was reduced in the low protein group (*P* < 0.05), while histidine supplementation failed to affect the liver and kidney weights (*P* > 0.05). Meanwhile, the relative weight of the spleen was not significantly affected in this study (Table 2).

### 3.3. Effect of Dietary Histidine on Serum Biochemical Indexes in Low Protein Diet-Fed Piglets

Results of serum biochemical indexes were shown in Table 3. Compared with the control group, serum alanine aminotransferase activity was markedly enhanced, and lactate dehydrogenase activity was reduced in the low protein group (*P* < 0.05). Although histidine exposure failed to alleviate the changes of alanine aminotransferase and lactate dehydrogenase activities, albumin abundance was markedly reduced in the histidine-supplemented group compared with that in the control (*P* < 0.05).

### 3.4. Effect of Dietary Histidine on Serum Amino Acid Pool in Low Protein Diet-Fed Piglets

Compared with the control group, serine, threonine, alanine, lysine, methionine, and tryptophan were markedly increased, and valine and isoleucine abundances were reduced in low protein diet-fed piglets (*P* < 0.05) (Table 4). Histidine supplementation markedly enhanced histidine and reversed tryptophan concentration (*P* < 0.05). Meanwhile, histidine tended to reversed the changes of methionine and valine, but the difference was insignificant (*P* > 0.05).

### 3.5. Effect of Dietary Histidine on Intestinal Morphology in Low Protein Diet-Fed Piglets

In the jejunum, piglets from the low protein group showed impaired intestinal morphology evidenced by the decreased trend of villus length, the ratio of villus to crypt depth, and villus surface area, which were slightly improved in the histidine treated group, but all these indexes were insignificant (*P* > 0.05) (Table 5).

In the ileum, villus length was significantly reduced in low protein diet-fed piglets (*P* < 0.05), while dietary supplementation with histidine failed to affect villus length and other morphologic indexes (*P* > 0.05) (Table 5).

### 3.6. Effect of Dietary Histidine on *α*-Diversity and Gut Microbiota Compositions in Low Protein Diet-Fed Piglets

Observed species, Shannon, Simpson, Chao1, ACE, and phylogenetic diversity were analyzed to evaluate the *α*-diversity of gut microbiota, while all of them were not markedly differentiated between three groups (Table 6), indicating that protein restriction and histidine treatment had little effect on *α*-diversity of gut microbiota in the current study.

Microbiota compositions at the phylum and genus levels were analyzed (Table 7). At the phylum level, *Firmicutes* and *Bacteroidetes* were two major bacteria and occupied up to 40%, but all of them were not markedly changed in response to low protein diet and histidine supplementation (*P* > 0.05). The relative abundance of *Spirochaetes* was markedly increased in low protein diet-fed piglets (*P* < 0.05) but not altered at the histidine group (*P* > 0.05). In addition, *Proteobacteria*, *Melainabacteria*, and *Tenericutes* were also tested at the ileum, but we failed to notice any significant difference between three groups.

At the genus level, *Succinivibrio*, *Anaerovibrio*, *Faecalibacterium*, *Alloprevotella*, *Lactobacillus*, *Butyrivibrio*, *Roseburia*, *Parabacteroides*, *Subdoligranulum*, *Oribacterium*, *Oscillospira*, *Streptococcus*, *Fournierella*, *Bacteroides*, and *Sphaerochaeta* were identified. Compared with the control group, the relative abundances of *Subdoligranulum* and *Oscillospira* were markedly reduced in response to dietary low protein diet (*P* < 0.05), while histidine exposure failed to alleviate the changes of *Subdoligranulum* and *Oscillospira* (*P* > 0.05). Interestingly, histidine-treated piglets exhibited higher abundances of *Butyrivibrio* and *Bacteroides* compared with the control and protein-restricted subjects (*P* < 0.05).

## 4. Discussion

Previous studies showed that dietary supplementation with amino acids improves growth performance in low protein diet-fed pigs. For example, supplementation of leucine in low protein diet improves protein deposition and meat quality in finishing pigs [12]. Also, the minimum standardized ileal digestible histidine to lysine ratio is suggested to be 28% in piglets to maintain growth performance by using the curvilinear-plateau model [13]. However, in this study, balancing dietary histidine compared with the control diet failed to affect final body weight and weight gain in pigs, while feed intake at week 6 was markedly enhanced in the histidine group. Furthermore, relative weight of organ and serum biochemical indexes were not altered in response to dietary histidine. The reason may be caused by the dietary dosage and time of histidine, and further studies are suggested to confirm the high histidine and long-term effect on the growth performance in low protein diet-fed piglets.

Our previous study revealed a marked reduction of muscle and serum histidine in low protein diet-fed pigs [3, 14]. Similarly, serum histidine concentration also tended to decrease in protein-restricted piglets, while balancing histidine in the diet markedly enhanced serum histidine, indicating that dietary supplementation with histidine enhanced intestinal absorption and circulating of histidine. Our previous studies reported that histidine and lysine exhibit competitive inhibitory association in transport and metabolism [11, 15], while dietary histidine in low protein diet failed to markedly inhibit lysine metabolism in the current study. Meanwhile, histidine supplementation markedly reversed tryptophan increase in low protein diet-fed piglets. Dietary tryptophan in pigs has been widely reported to improve growth performance and gut barrier [16, 17]; thus, we speculated the serum tryptophan concentration failed to correlate to growth performance in this study. Together, dietary supplementation with histidine enhanced serum histidine concentration and affected tryptophan metabolism in low protein diet-fed piglets, but the detailed mechanism should be further studied.

Growth performance of piglet is highly associated with intestinal functions (i.e., nutrient digestibility, absorption, and barrier function). Dietary amino acids are mainly used by intestinal epithelial cells to provide energy and form protein molecules [18, 19]. For example, our previous study showed that dietary supplementation with glutamate markedly improved intestinal morphological structure, intestinal barrier function, and antioxidant system in mycotoxin-challenged pigs [18]. However, dietary histidine slightly improved villus length, the ratio of villus to crypt depth, and villus surface area in the jejunum. Jiang et al. reported that histidine supplementation in diets (3.7 up to 12.2 g/kg) blocked Cu-induced decreases in intestinal tight junction proteins and mRNA levels of antioxidant genes [20]. Thus, the effect of histidine on intestinal morphology should be further investigated in different dosages and models.

Dietary changes and supplements are highly associated with gut microbiota, which further affect host metabolism. For example, intestinal bacterial diversity and the relative abundances of *Actinobacteria*, *Saccharibacteria*, and *Synergistetes* at the phylum level were increased in a pig model when feeding a low diet [11]. In addition, gut microbiota alteration contributed to host metabolism, such as energy metabolism, lipid metabolism, and carbohydrate metabolism [21]. In this study, we found that histidine affected tryptophan metabolism, while tryptophan metabolite (melatonin) markedly improved gut microbiota evidenced by increasing bacterial diversity and affecting the relative abundances of *Bacteroides* and *Alistipes* in murine model [21]. The current results showed that dietary histidine failed to affect bacterial diversity, but histidine-treated piglets exhibited higher abundances of *Butyrivibrio* and *Bacteroides* compared with the control and protein-restricted subjects. *Butyrivibrio* has been identified in pigs with high residual feed intake [22], indicating that *Butyrivibrio* may involve in feed intake regulation. *Bacteroides* species are significant clinical pathogens and are found in most anaerobic infections [23]. However, the mechanism of histidine-mediated Bacteroides proliferation and its role in piglet were not understood in the current study.

In conclusion, low protein diet reduced growth performance and affected serum amino acids and gut microbiota. Dietary supplementation with histidine markedly enhanced serum histidine concentration in low protein diet-fed piglets and restored tryptophan level. In addition, histidine-treated piglets exhibited higher abundances of *Butyrivibrio* and *Bacteroides*.

## Figures and Tables

**Table 1:** Effect of dietary histidine on growth performance in low protein diet-fed piglets.

Item	Control group	Low protein group	Histidine group	Sem	*P* value
Initial body weight (kg)	7.69	7.64	7.68	0.17	0.99
Final body weight (kg)	21.53^a^	16.86^b^	16.51^b^	0.69	<0.01
Average daily weight gain (kg)	0.39^a^	0.26^b^	0.25^b^	0.02	<0.01
Average daily feed intake (kg)					
Week 1	0.42	0.41	0.39	0.01	0.18
Week 2	0.66	0.64	0.53	0.03	0.16
Week 3	0.61	0.51	0.54	0.04	0.51
Week 4	0.63^a^	0.47^b^	0.45^b^	0.03	<0.01
Week 5	0.94^a^	0.64^b^	0.63^b^	0.04	<0.01
Week 6	0.85^ab^	0.69^b^	0.87^a^	0.04	0.08
Week 7	1.11^a^	0.75^b^	0.80^b^	0.05	<0.01

^a,b^Within a row means the difference was significant with different superscripts (*P* < 0.05).

**Table 2:** Effect of dietary histidine on relative organ weight in low protein diet-fed piglets.

Item	Control group	Low protein group	Histidine group	Sem	*P* value
Liver	2.71^b^	3.00^a^	3.05^a^	0.06	0.05
Kidney	0.53^a^	0.45^b^	0.49^b^	0.01	<0.01
Spleen	0.19	0.20	0.21	0.01	0.55

^a,b^Within a row means the difference was significant with different superscripts (*P* < 0.05).

**Table 3:** Effect of dietary histidine on serum biochemical indexes in low protein diet-fed piglets.

Item	Control group	Low protein group	Histidine group	Sem	*P* value
Total protein	55.43	54.59	52.95	0.89	0.52
Albumin	39.69^a^	37.23^ab^	33.99^b^	0.82	0.01
Alanine aminotransferase	60.44^b^	78.60^a^	75.20^a^	2.90	0.02
Aspartate aminotransferase	72.43	62.25	69.13	3.28	0.46
Alkaline phosphatase	291.86	263.50	269.00	8.97	0.43
Gamma glutamyl transpeptidase	46.50	40.00	44.63	1.79	0.34
Lactate dehydrogenase	923.38^a^	722.38^b^	733.50^b^	34.76	0.02
Acid phosphatase	18.52	20.15	18.28	1.61	0.89
NH3L	287.80	295.36	275.86	16.56	0.89
Immunoglobulin M	0.31	0.26	0.29	0.01	0.26
Cholinesterase	711.75	727.50	672.75	20.22	0.54

^a,b^Within a row means the difference was significant with different superscripts (*P* < 0.05).

**Table 4:** Effect of dietary histidine on serum amino acid pool in low protein diet-fed piglets.

Item	Control group	Low protein group	Histidine group	Sem	*P* value
Histidine	2.32^ab^	2.04^b^	2.82^a^	0.13	0.05
Serine	15.21^b^	20.59^a^	19.61^a^	0.74	<0.01
Arginine	29.77	28.10	27.47	0.99	0.64
Glycine	115.42	103.13	100.51	3.64	0.20
Aspartic acid	3.46	2.76	2.65	0.18	0.14
Glutamic acid	66.34	61.76	57.20	2.73	0.41
Threonine	22.90^b^	95.46^a^	94.13^a^	9.61	<0.01
Alanine	80.23^b^	109.29^a^	104.04^a^	5.15	0.04
Proline	34.44	37.20	37.87	1.53	0.63
Cysteine	0.79	0.59	0.74	0.08	0.56
Lysine	42.97^b^	81.43^a^	77.76^a^	5.61	<0.01
Tyrosine	15.40	14.42	14.43	0.51	0.70
Methionine	15.22^b^	21.83^a^	17.06^ab^	1.30	0.09
Valine	10.95^a^	8.51^b^	9.52^ab^	0.41	0.05
Isoleucine	12.44^a^	4.30^b^	5.21^b^	0.85	<0.01
Leucine	19.80	18.19	18.04	0.63	0.47
Phenylalanine	10.47	10.85	11.13	0.28	0.64
Tryptophan	11.47^b^	14.6^a^	11.81^b^	0.58	0.05

^a,b^Within a row means the difference was significant with different superscripts (*P* < 0.05).

**Table 5:** Effect of dietary histidine on intestinal morphology in low protein diet-fed piglets.

Item	Control group	Low protein group	Histidine group	Sem	*P* value
Jejunum					
Villus length	513.52	469.42	542.06	14.52	0.11
Villus width	139.61	142.18	135.31	3.77	0.76
Crypt depth	202.84	194.10	201.17	5.40	0.80
Villus length/crypt depth	2.67	2.37	2.73	0.11	0.35
Villus surface area (mm^2^)	0.24	0.21	0.23	0.01	0.23
Ileum					
Villus length	361.30^a^	307.13^b^	313.07^b^	7.72	<0.01
Villus width	104.52	109.24	95.82	2.73	0.08
Crypt depth	121.09	111.34	109.65	3.95	0.52
Villus length/crypt depth	2.75	2.81	2.86	0.10	0.91
Villus surface area (mm^2^)	0.11	0.10	0.10	0.00	0.27

^a,b^Within a row means the difference was significant with different superscripts (*P* < 0.05).

**Table 6:** Effect of dietary histidine on *α*-diversity of gut microbiota in low protein diet-fed piglets.

Item	Control group	Low protein group	Histidine group	Sem	*P* value
Observed species	594.88	595.75	589.00	9.43	0.96
Shannon	6.60	6.66	6.64	0.09	0.97
Simpson	0.97	0.97	0.98	0.00	0.46
Chao1	657.03	663.29	636.86	12.81	0.70
ACE	658.76	662.57	628.47	12.07	0.47
Phylogenetic diversity	45.76	45.15	45.37	0.67	0.40

^a,b^Within a row means the difference was significant with different superscripts (*P* < 0.05).

**Table 7:** Effect of dietary histidine on gut microbiota compositions in low protein diet-fed piglets.

Item	Control group	Low protein group	Histidine group	Sem	*P* value
Phylum level (%)					
Firmicutes	50.53	47.44	53.30	1.26	0.17
Bacteroidetes	43.52	41.88	38.71	1.08	0.19
Proteobacteria	2.45	4.38	3.72	0.61	0.44
Spirochaetes	0.63^b^	2.86^a^	2.63^a^	0.39	0.03
Melainabacteria	0.85	0.41	0.28	0.12	0.15
Tenericutes	0.44	0.51	0.43	0.06	0.88
Genus level (%)					
Succinivibrio	1.61	0.52	1.85	0.42	0.56
Anaerovibrio	1.72	2.20	3.98	0.75	0.45
Faecalibacterium	2.41	1.25	1.84	0.28	0.26
Alloprevotella	1.84	2.38	1.55	0.31	0.54
Lactobacillus	2.58	1.06	1.59	0.40	0.31
Butyrivibrio	0.01^b^	0.05^b^	0.56^a^	0.11	0.06
Roseburia	0.66	1.19	0.94	0.20	0.56
Parabacteroides	0.52	0.83	1.05	0.11	0.11
Subdoligranulum	1.13^a^	0.29^b^	0.31^b^	0.13	<0.01
Oribacterium	0.31	0.24	0.02	0.07	0.22
Oscillospira	1.56^a^	0.92^b^	0.96^b^	0.13	0.06
Streptococcus	0.24	0.15	0.06	0.04	0.18
Fournierella	0.83	0.40	0.39	0.10	0.10
Bacteroides	0.09^b^	0.27^b^	0.74^a^	0.10	0.02
Sphaerochaeta	0.39	0.84	0.50	0.11	0.24

^a,b^Within a row means the difference was significant with different superscripts (*P* < 0.05).

## Data Availability

The data used to support to the findings of this study are available from the corresponding author upon request.
